# Lift-Off Assisted Patterning of Few Layers Graphene

**DOI:** 10.3390/mi10060426

**Published:** 2019-06-25

**Authors:** Alessio Verna, Simone Luigi Marasso, Paola Rivolo, Matteo Parmeggiani, Marco Laurenti, Matteo Cocuzza

**Affiliations:** 1 Chilab—Materials and Microsystems Laboratory, DISAT, Politecnico di Torino—Via Lungo Piazza d’Armi 6, IT 10034 Chivasso (Torino), Italy; simone.marasso@polito.it (S.L.M.); paola.rivolo@polito.it (P.R.); matteo.parmeggiani@polito.it (M.P.); marco.laurenti@polito.it (M.L.); matteo.cocuzza@infm.polito.it (M.C.); 2 CNR-IMEM, Parco Area delle Scienze 37a, IT 43124 Parma, Italy; 3 Center for Sustainable Future Technologies, Italian Institute of Technology, Via Livorno 60, IT 10144 Torino, Italy

**Keywords:** graphene, patterning, Pt, 2D materials, chemical vapor deposition (CVD)

## Abstract

Graphene and 2D materials have been exploited in a growing number of applications and the quality of the deposited layer has been found to be a critical issue for the functionality of the developed devices. Particularly, Chemical Vapor Deposition (CVD) of high quality graphene should be preserved without defects also in the subsequent processes of transferring and patterning. In this work, a lift-off assisted patterning process of Few Layer Graphene (FLG) has been developed to obtain a significant simplification of the whole transferring method and a conformal growth on micrometre size features. The process is based on the lift-off of the catalyst seed layer prior to the FLG deposition. Starting from a SiO_2_ finished Silicon substrate, a photolithographic step has been carried out to define the micro patterns, then an evaporation of Pt thin film on Al_2_O_3_ adhesion layer has been performed. Subsequently, the Pt/Al_2_O_3_ lift-off step has been attained using a dimethyl sulfoxide (DMSO) bath. The FLG was grown directly on the patterned Pt seed layer by Chemical Vapor Deposition (CVD). Raman spectroscopy was applied on the patterned area in order to investigate the quality of the obtained graphene. Following the novel lift-off assisted patterning technique a minimization of the de-wetting phenomenon for temperatures up to 1000 °C was achieved and micropatterns, down to 10 µm, were easily covered with a high quality FLG.

## 1. Introduction

In the last years, Single Layer or Few Layer Graphene (SLG-FLG) have been widely exploited to obtain novel high performance devices for different types of applications from electronics [1] to energy storage [2], sensors [3], biomedical implants [4] and others [5,6]. The quality of the SLG or FLG has been found to be a critical issue for the functionality of the devices and hence it is fundamental to improve the production and synthesis steps to avoid defects. To develop an SLG and FLG based device, the typical technological processes involved are: Chemical Vapor Deposition (CVD) on metal catalyst seed layers such as Cu or Ni foils [6]; the transferring step on polymer,—that is, poly(methyl methacrylate) (PMMA)—by spin coating and wet etching; the deposition on active regions, that is, metallic electrodes on SiO_2_ finished Si substrates; and finally, the patterning step by plasma etching, laser ablation or other techniques [7]. The transfer of graphene onto arbitrary substrates is generally accomplished by polymer-assisted procedures. The transfer process consists of removing graphene from the growing substrate with the aid of a sacrificial polymer layer. For this purpose, several polymers like poly(methyl methacrylate) and polyvinylidene fluoride (PVDF) are widely employed as sacrificial supports [7,8]. Removing of the growing catalyst substrate is accomplished by chemical etching [9] or by electrochemical delamination (ED) [10]. Finally, the graphene/polymer self-standing membrane is attached on the target substrate and the polymer is then removed with a suitable solvent [8]. Despite being widely used, this approach may lead to the formation of undesirable defects and damages within the graphene layer. Hence, a major challenge is to minimize such defects by contact transfer processes at wafer scale [11]. As an alternative, the direct growth of graphene on insulating substrates using Cu vapor [12] or ultrathin Cu [13,14] and Ni [15] films as catalyst has been attempted; however, these methods do not allow for a precise control of the defects. Another interesting approach is to pattern the graphene directly on Cu substrates before transfer [16] ensuring a precise reproduction of the pattern but involving time consuming polymer transfer. Due to the difficulties to grow graphene directly on Cu or Ni thin films and due to the incompatibility of these metals in most biological/electrochemical applications, usually graphene is transferred on Au or Pt electrodes exploiting the previously described methods.

Several applications require the use of Pt electrodes coated with graphene. These include atomic force microscope (AFM) tips for nanoscale electrical characterization [17], friction reduction in Micro Electro Mechanical System (MEMS) [18], counter electrodes for dye-sensitized solar cells [19], microelectrodes for neurostimulation [20], amperometric sensors in electrophoresis devices [21], electrodes for the electrochemical adsorption of dyes by cyclic voltammetry [22] and electrodes decorated with Pt nanoparticles for electrochemical applications [23,24]. Furthermore, graphene patterning on this type of electrodes is crucial to define the active area of graphene on devices like sensors [3,25,26]. This step introduces further defects and damages on the edges [27] or needs for additional technological processes [28].

Due to the high melting point (T_m_ = 1768 °C) and low vapor pressure (1.3 × 10^−14^ mmHg), Pt is a well-known catalyst for growing graphene by CVD [29]. In fact, it has been demonstrated that sputtered Pt thin films do not suffer from de-wetting issues, typical of Cu and Ni, during monolayer or few layers graphene growth [30]. The stability of Pt thin films against de-wetting [31] phenomena at high temperature on Si/SiO_2_ wafers can be also tuned and improved by increasing the thickness of the film [30]. In addition, the use of adhesion layers may lead to similar results with thinner films as well as e-beam evaporated films. Usual adhesion layers for Pt are metals like Ti, Ta or Cr. However, their exposure to high temperatures causes inter-diffusion or oxide formation and their subsequent degradation [32]. Alumina (Al_2_O_3_) is preferred as Pt adhesion layer in high temperature applications [33] due to its thermal and chemical stability. Moreover, it can be deposited with common techniques such as sputtering or e-beam evaporation [34] and then easily integrated in Pt deposition process. Finally, Pt is also an optimal choice for electrodes in chemical/biological sensors [35] as well as for high temperature micro-hotplates sensors [36,37] due to its high chemical and temperature stability. Therefore, the direct growth of high quality graphene on patterned Pt thin films may represent an advantage and simplification of the entire device fabrication process.

Here, the direct growth of CVD FLG on Pt thin film was obtained by a lift-off assisted patterning. Al_2_O_3_ was used as adhesion layer to avoid de-wetting of Pt film. FLG was grown on patterned Al_2_O_3_/Pt substrates with features down to 10 µm. The graphene quality on patterned areas was evaluated and compared to the graphene grown on un-patterned film by Raman analysis.

## 2. Materials and Methods

### 2.1. Lift-Off Assisted Patterning

**Figure 1:**
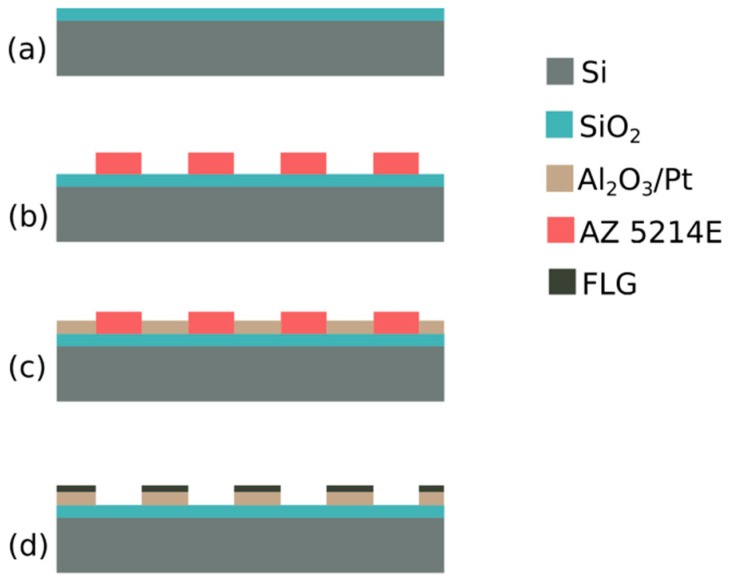
Process flow: (**a**) starting substrate, (**b**) photolithography, (**c**) Al_2_O_3_/Pt deposition and lift-off, (**d**) graphene growth.

The proposed novel method is based on the lift-off of the catalyst seed layer prior to the FLG deposition (Figure 1). Single side polished, P type, (100) silicon wafers (resistivity 1–10 Ω·cm) finished with 1 µm thermal oxide (supplied by Si-Mat, Kaufering, Germany) were employed for the patterning process. Two cm × two cm squares samples were used in order to fit into the graphene deposition system, which was the NANOCVD-8G system from Moorfield Nanotechnology Ltd. (Cheshire, UK). Samples were cleaned in an acetone bath, rinsed with isopropyl alcohol and then patterned using image reversal photoresist (Microchemicals AZ 5214E, Ulm, Germany) and standard UV (ultraviolet) lithography, through a photomask. The next step was the deposition of 30 nm of Al_2_O_3_ (purity 99.99%) followed by 60 nm of Pt (purity 99.99%) by electron beam evaporation (ULVAC EBX-14D, Chigasaki, Japan) with a deposition rate of 2–3 Å/s, both for Al_2_O_3_ and Pt, in high vacuum (<10^−5^ mTorr) and heating the samples at 150 °C during the deposition process.

After the deposition, the photoresist was stripped with dimethyl sulfoxide (DMSO) at 50 °C and the samples were rinsed with deionized water (DI) and dried with nitrogen.

### 2.2. Graphene Deposition on Pt Film

Graphene was grown on Si/SiO_2_/Al_2_O_3_/Pt substrates by a cold-wall chemical vapor deposition (CVD) reactor operating at low pressure. To remove contaminants from the surface, a two-step annealing of the substrates was performed: 2 min at 900 °C under reducing flow of Ar at 190 sccm and H_2_ at 10 sccm (flow control regime) and then 30 s at 1000 °C in an atmosphere composed of Ar 90% and H_2_ 10% at 10 torr (pressure control regime). The growth of graphene was then carried out at 1000 °C for 300 s, in a mixed atmosphere of Ar (80%), H_2_ (10%) and CH_4_ (10%) at 10 torr. The samples were finally cooled down to 200 °C under a reducing flow of Ar + H_2_ (190 sccm and 10 sccm) and then to room temperature in Ar atmosphere.

### 2.3. Characterization

Un-patterned Al_2_O_3_/Pt thin films were characterized with field emission scanning electron microscopy (FESEM) after an annealing treatment at 900 °C, 1000 °C and 1050 °C to investigate the high temperature effect related to the CVD graphene growth process. Images were obtained with FESEM ZEISS Supra 40 (Oberkochen, Germany). For this purpose, the annealing was performed in the same atmosphere and time duration previously described for the growth of graphene but excluding CH_4_ in the gas mixture.

X-Ray Diffraction (XRD) was performed on un-patterned Si/SiO_2_/Al_2_O_3_/Pt substrates with the twofold aim of analysing the corresponding crystal structure and orientation and evaluating the effect of the thermal annealing at 1000 °C. XRD patterns were collected using a Panalytical X’Pert Diffractometer (PANalytical, Almelo, The Netherlands) in Bragg-Brentano configuration, equipped with a Cu Kα radiation as X-ray source (λ = 1.54059 Å).

Pt/FLG substrates were characterized by means of a Renishaw InVia Reflex micro-Raman spectrometer (Renishaw plc, Wottonunder-Edge, UK), equipped with a cooled CCD camera. The Raman source was a laser diode (λ = 514.5 nm) and samples inspection occurred in backscattering light collection through a 50× microscope objective for all the single spectra acquisition. The spectra of the patterned structures were obtained by focusing the laser spot on their centre, while a Raman map of the 10 µm-wide circle was collected by scanning, by means of a long working distance 100× objective, a 16 µm × 16 µm area, with a 0.5 µm step. The spectral map analysis was performed by means of the Renishaw WiRE 3.2 software. To collect both the single spectra and the map, 50 mW laser power, 60 s of exposure time and 4 accumulations were employed.

Optical images were acquired with a Nikon Eclipse ME600 microscope (Nikon, Tokyo, Japan).

## 3. Results

**Figure 2:**
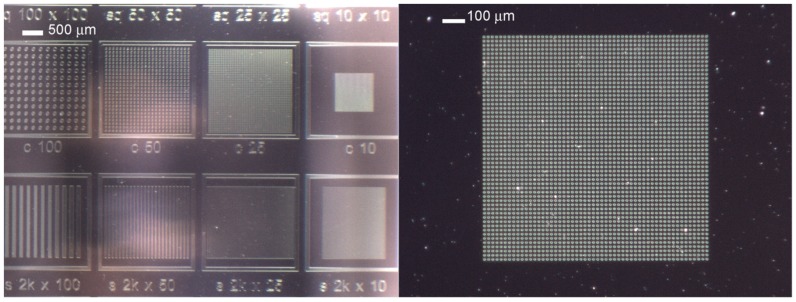
Lift-off assisted patterning of few layers graphene (FLG) on Pt/Al_2_O_3_ catalyst: optical images of the patterned catalyst with different sizes features.

Lift-off assisted patterning of FLG has been successfully obtained (Figure 2) on Al_2_O_3_/Pt catalyst film.

### 3.1. Morphologica Characterization of De-Wetting Dynamic

**Figure 3:**
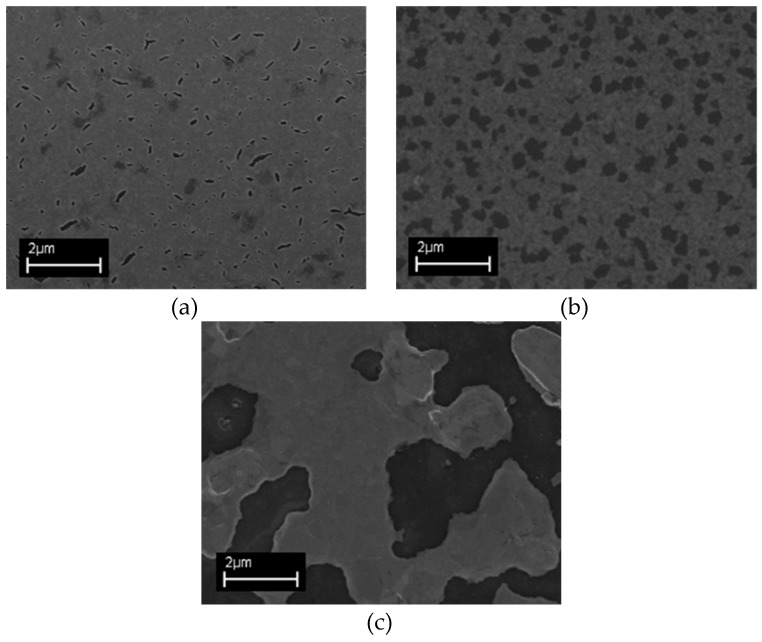
Comparison of field emission scanning electron microscope (FESEM) images of Pt/Al_2_O_3_ annealed at 900 °C (**a**), 1000 °C (**b**) and 1050 °C (**c**). The film becomes highly discontinuous at 1050 °C although de-wetting process starts below 900 °C.

The temperature effects were evaluated by thermal annealing tests on Al_2_O_3_/Pt layer at 900 °C, 1000 °C and 1050 °C (Figure 3).

FESEM images demonstrate that the Al_2_O_3_ adhesion layer allows for controlling the de-wetting process (see supplementary information) to achieve a FLG growth temperature up to 1000 °C. It can be noticed that a detrimental effect appears at 1050 °C, where a discontinuous film is formed, making it impractical to use for most technological applications.

### 3.2. XRD Characterization

**Figure 4:**
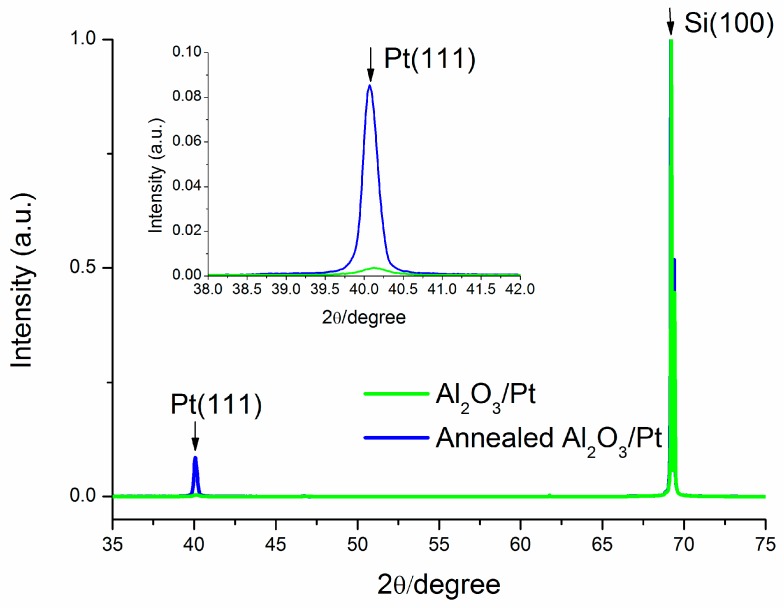
X-ray diffraction (XRD) patterns of Al_2_O_3_/Pt samples, before and after annealing at 1000 °C. Annealed sample shows the amplification of Pt(111) phase which is suitable for graphene growth. The inset shows a magnification of the Pt(111) phase.

The diffraction spectrum obtained by XRD investigation (Figure 4) shows a comparison between as-grown Al_2_O_3_/Pt samples and the 1000 °C annealed one. Apart from the contribution coming from the Si substrate (2θ–69.2°), a single diffraction peak is detected at 40.1° in both cases and ascribed to the family of Pt(111) crystal planes (JCPDS Card 04-0802). After annealing, the crystal quality of the Pt layers turns out to be improved, as demonstrated by the higher Pt(111) peak intensity. Moreover, the (111) crystal orientation is also highly desirable for promoting graphene growth [23]. This characterization validates Al_2_O_3_ as adhesion layer for this kind of application; indeed, with respect to previous work on a similar process [31], Al_2_O_3_ prevents a premature de-wetting for e-beam evaporated Pt film.

### 3.3. Raman Characterization of Patterned Pt

The patterned Al_2_O_3_/Pt was characterized by Raman spectroscopy to evaluate the quality of the grown graphene, which according to Wang et al. was about 2–3 layers [38].

**Figure 5:**
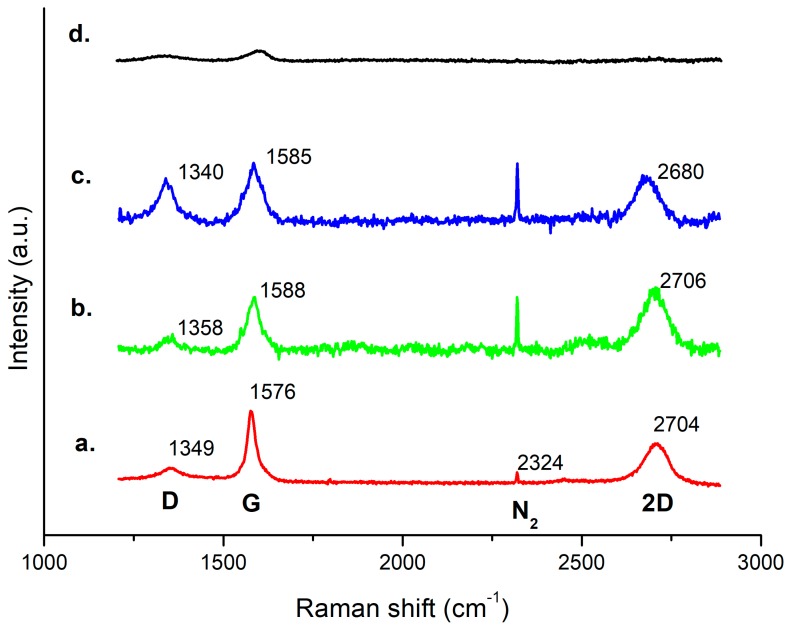
Comparative Raman spectra of un-patterned Pt/FLG (**a**), 100 µm strip pattern (**b**), 5 µm strip pattern (**c**) and blank silicon collected between two 100 µm Pt strips (**d**). The main peaks labels are shown.

The analysis was performed with the aim to evaluate the selective growth of FLG on Pt patterns with respect to SiO_2_ and the correlation between the FLG defectivity and the patterns sizes. Furthermore, growing temperature effect was investigated. Raman spectra of FLG on both un-patterned (red curve) and patterned Pt samples ranging from 5 to 100 µm wide strips were reported (Figure 5). For the patterned Pt samples, the Raman spectra were collected on a 1–2 µm wide area, far from the edges. The intensity (I), position and shape of D, G and 2D peaks (centred at ~1350, 1580 and 2700 cm^−1^ respectively) are similar for the 100 µm patterned and un-patterned areas but the intensity of the D peak differs on the 5 µm pattern. The presence of an ubiquitous peak at ~2324 cm^−1^ can be related to atmospheric N_2_ gas fundamental vibration-rotation as previously reported [39].

For the FLG growth on the un-patterned Pt sample, the G peak only differs from the patterned ones in shape and intensity with respect to the D band: a narrower and more symmetric band and an I_D_/I_G_ ratio of ~0.20 are observable. Moreover, the calculated I_D_/I_G_ is ~0.31 on the 100 µm pattern and ~0.73 on the 5 µm pattern suggesting that the presence of the microstructures could induce a more disordered superposition of the graphene few layers. As pointed out in the review by Ferrari and Basko [40], other factors confirm the disorder induced in the case of patterned Pt/FLG with respect to plain film; these include: the increased dispersion of the G peak; the small elbow at the right of the G peak which could be associated with a small D’ peak and the noise at the left of 2D which could be associated with the D’’ peak. On the other hand, the shape and position of the 2D band for both samples (un-patterned and patterned) indicate that the number and the quality of the deposited graphene sheets are quite comparable, as the peak, though symmetric, cannot be fitted by one Lorentzian and it has a FWHM of ~77 cm^−1^, 64 cm^−1^ and 84 cm^−1^ respectively for un-patterned, 100 µm and 5 µm patterns, which are compatible with the characteristics of FLG grown on a nickel-coated SiO_2_/Si substrate, previously reported by Park et al. [41].

**Figure 6:**
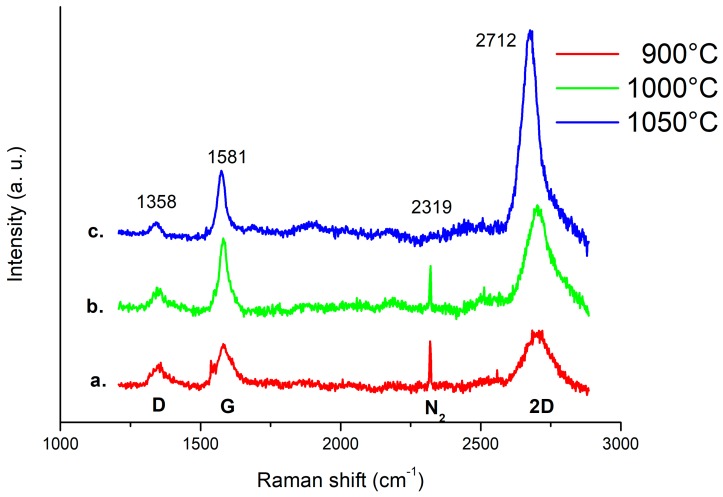
Comparative Raman spectra of 100 µm patterns of Pt/FLG grown at 900 °C (**a**), at 1000 °C (**b**) and at 1050 °C (**c**). The ratio between 2D and G peaks intensities increases indicating a reduction in the number of layers with temperature. The main peaks labels are shown.

Patterned Pt/FLG grown at 900 °C, 1000 °C and 1050 °C was further characterized by Raman spectroscopy (Figure 6). It can be noticed that at 1050 °C graphene quality improves although Pt thin film undergoes de-wetting effect and, with the increasing temperature, the ratio between 2D and G peaks also increases indicating a reduction in the number of layers. Moreover, both D peak intensity reduction and G peak sharpness indicate a minimization of the graphene defects. But from the comparison with morphological analysis (Figure 3), at 1050 °C the de-wetting of the Pt film has relevant detrimental effect.

**Figure 7:**
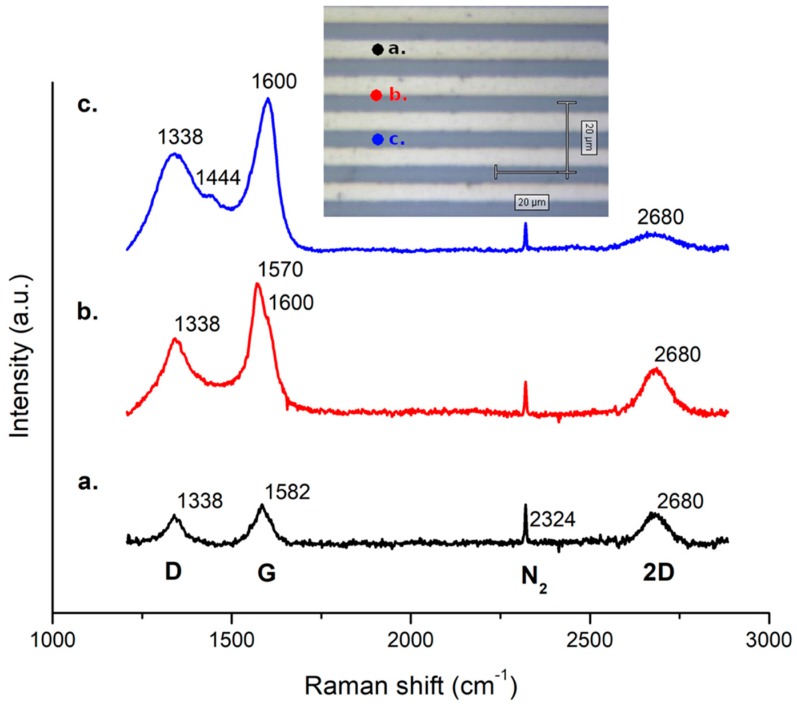
Raman spectra on 5 µm wide strips at different positions at the centre of the strip (**a**), on the border (**b**) and between two strips (**c**). The main peaks labels are shown. The inset shows an optical image of the sample. A graphitic carbon residual is observed in (**c**) while in un-patterned areas (Figure 5d) no carbon residuals are present. This shows that amorphous carbon deposition is catalysed by the presence of platinum also in a halo of 1–2 µm outside the pattern.

Figure 7 shows FLG Raman spectra from the centre of a 5 µm strip to the border of the same pattern and then in a region 2.5 µm far from the edge. A transition from graphene to graphitic carbon residual is observed as the developing of D and G peaks indicate the presence of sp^2^ carbon with a consistent number of defects as previously reported [42].

**Figure 8:**
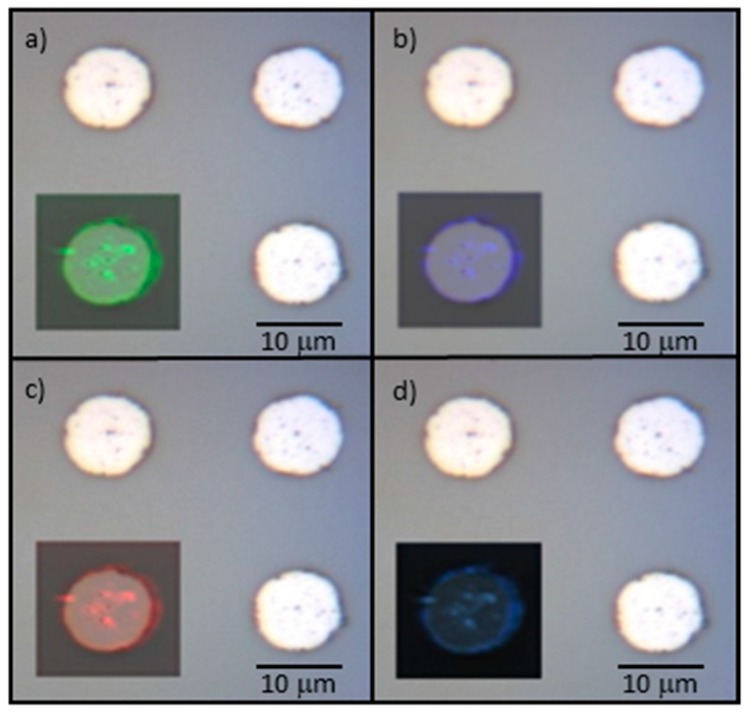
Raman map of a 10 µm wide circle. The map is superimposed to the optical image of 4 circles pattern for comparison. Map shows intensities distribution at 1350 cm^−1^ (**a**), 1580 cm^−1^ (**b**), 2700 cm^−1^ (**c**), overlap of all the selected Raman shift intensity distributions (**d**).

In order to verify the homogeneity of the FLG distribution and possible physical boundary effects, the scanning of a 16 × 16 µm^2^ area, including circle-shaped patterns (10 µm in diameter), was performed. The collected spectral Raman map (Figure 8) highlights that, in the inner region of the microstructure, the intensities of the D, G and 2D bands are quite constant in distribution and mutual ratio. Then, all the peak intensities increase by approaching the edge of the micro-circle, suggesting an accumulation of more defective and lower quality graphene sheets within such regions. Beyond the microstructure boundaries, no Raman features related to FLG are present, in accordance with blank spectrum (black curve) of Figure 6. This demonstrates the high selectivity of the growing process. Regarding defects accumulation on the edges, it is possible to assume that the discontinuities on the catalyst can affect the formation of graphene crystal domains thus leading to a more disordered growth.

## 4. Discussion

The reported analysis demonstrates that the lift-off assisted patterning is a valid method to obtain good quality FLG on Pt layer. A significant time reduction with respect to traditional process was achieved since the typical transferring steps were completely skipped. In addition, this method is not affected by the contamination of supporting polymers as PMMA. The obtained optimal repeatability on micrometric patterns allows for covering Pt film with every layouts and, more important, Pt can be deposited with common techniques such as sputtering or e-beam evaporation and then easily integrated in a full device fabrication process. Pt represents an optimal metal selection for electrodes in chemical/biological sensors [5] as well as for high temperature micro-hotplates in Micro Electro Mechanical System (MEMS) [6], due to its high chemical and temperature stability and hence the implementation of this method is of high relevance for a wide range of applications from biosensing to neuronal stimulation.

## 5. Conclusions

This FLG was grown directly on the patterned Pt seed layer by Chemical Vapor Deposition (CVD). The use of a proper adhesion layer, Al_2_O_3_, for the Pt film allows for raising the FLG growth temperature up to 1000 °C. The lift-off process of the catalyst, obtained by a standard photolithographic step, leads to a significant time reduction and consequent costs, of the graphene patterning since the typical transferring and etching steps were completely skipped, moreover an optimal repeatability on micrometric patterns can be easily obtained. The Raman characterization shows that the micropatterning was effective, and an accumulation of defects was mostly observed on the edges due to the discontinuity of the patterns. Since Pt is one of the most used materials for electrochemical or gas sensors due to its high thermal and chemical stability, the presented patterning approach has a potential high impact on the fabrication of graphene-based devices, when high quality graphene is required on noble metal electrodes. Moreover, the presented process can be applied to fabricate microelectrodes directly decorated with graphene on a whole wafer of any size avoiding the constraints correlated to polymer-assisted graphene transfer and etching.

## Supplementary Materials

The following are available online at https://www.mdpi.com/2072-666X/10/6/426/s1, Figure S1: FESEM images at different magnifications of graphene growth on Pt at 900 °C. Figure S2: FESEM images at different magnifications of graphene growth on Pt at 1000 °C. Figure S3: FESEM images at different magnifications of graphene growth on Pt at 1050 °C. Table S1: Percentage of Pt coverage to evaluate de-wetting.
