# Supplementary material for: Lift-Off Assisted Patterning of Few Layers Graphene
Source: Micromachines (Basel). 2019 Jun 25;10(6):426. doi: 10.3390/mi10060426 (PMC6631601; doi:10.3390/mi10060426)
Supplement: Supplementary file 1 [file micromachines-10-00426-s001.pdf]

Supplementary Materials: Lift-Off Assisted Patterning of Few Layers Graphene

Alessio Verna, Simone Luigi Marasso, Paola Rivolo, Matteo Parmeggiani, Marco Laurenti and Matteo Cocuzza

The effects of Al_2O_3/Pt film de-wetting were investigated before FLG growth. The behavior of de-wetting process has been studied at 900°C, 1000°C and 1050°C.

Morphological Analysis

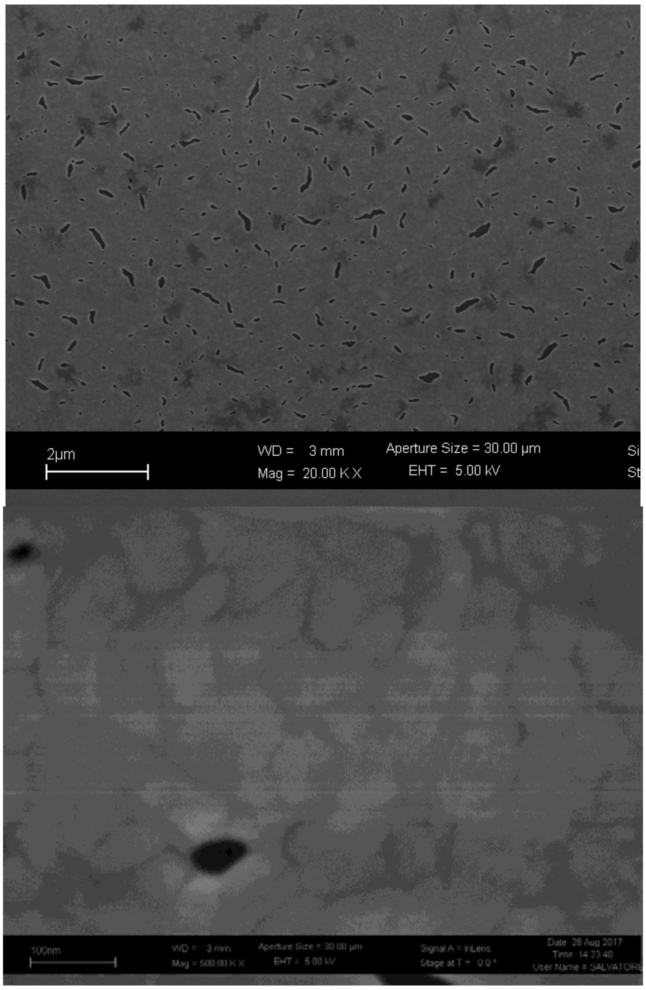

Figure S1. FESEM images at different magnifications of graphene growth on Pt at 900 °C.

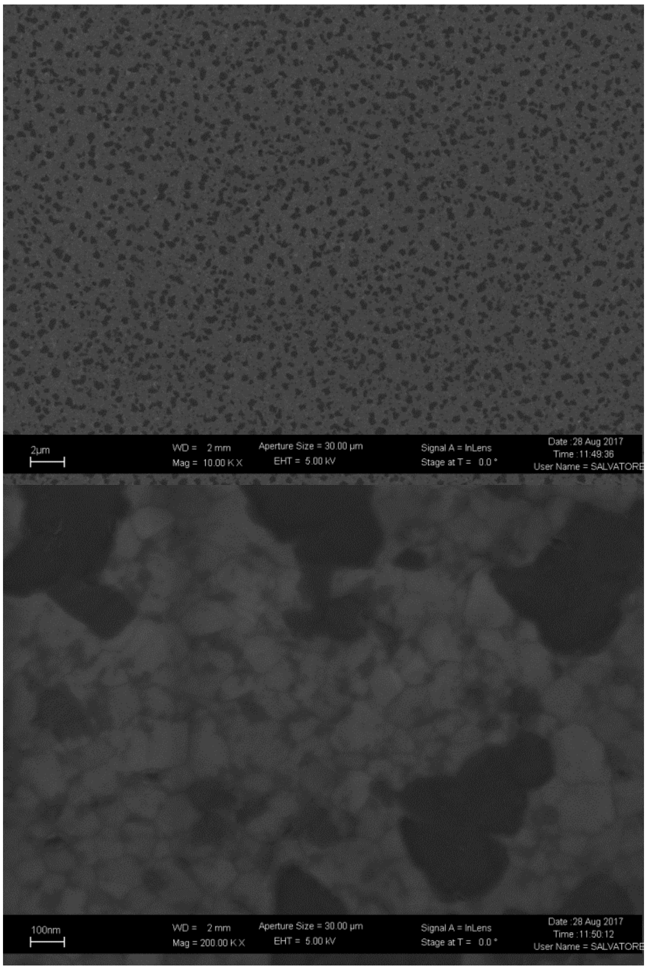

Figure S2. FESEM images at different magnifications of graphene growth on Pt at 1000 °C.

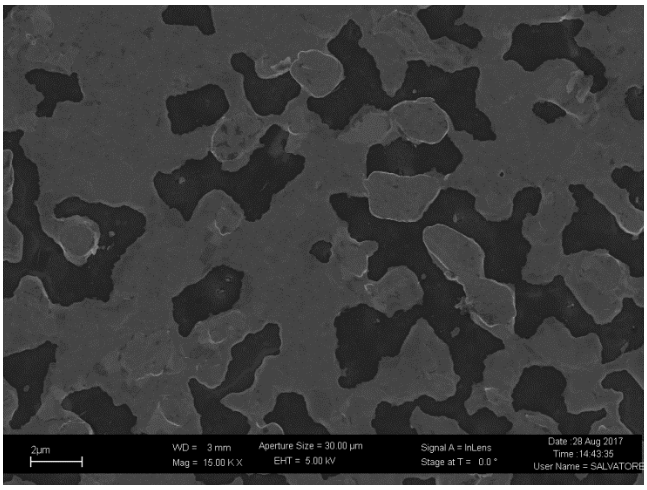

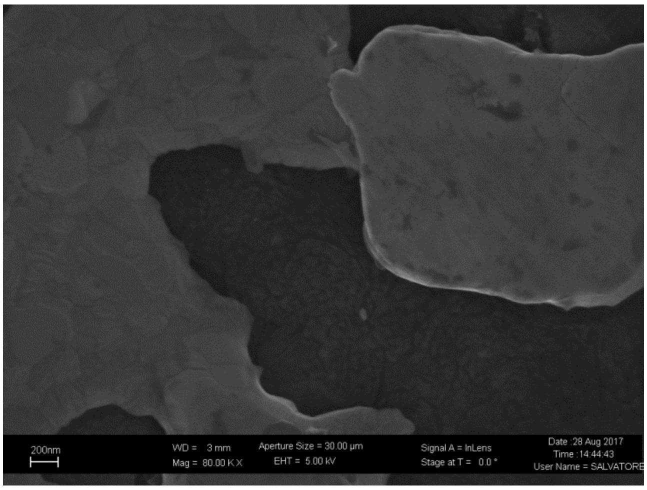

Figure S3. FESEM images at different magnifications of graphene growth on Pt at 1050 °C.

Graphical Evaluation of De-Wetting Dynamic

Table S1. Percentage of Pt coverage to evaluate de-wetting.

Temperature (°C)	FESEM Image	Threshold	% Pt
900	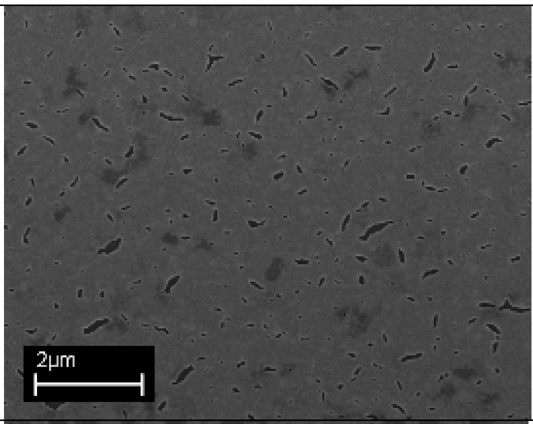	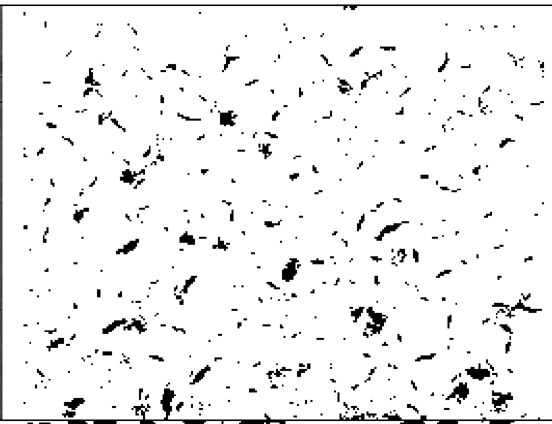	95.2
1000	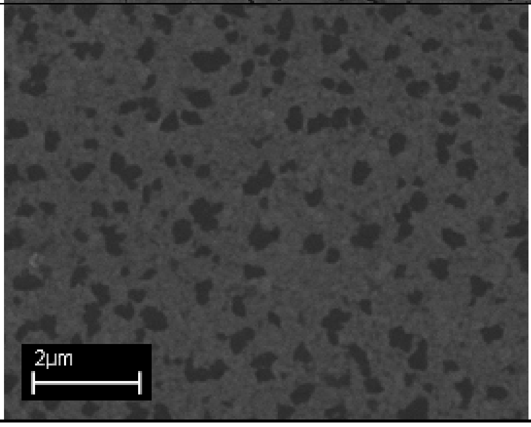	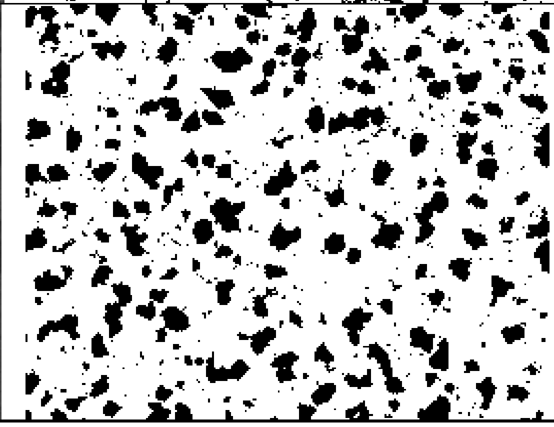	77.2

1050	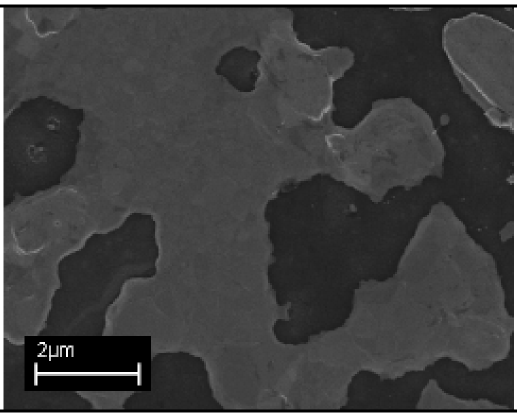	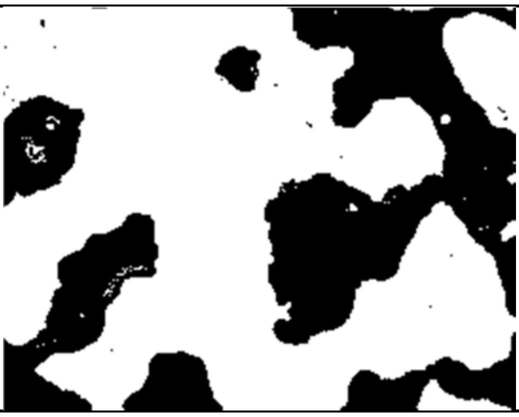	65.3

Threshold was automatically calculated with image manipulation software GIMP (threshold at the mean value of the histogram). The percentage of Pt coverage was calculated as mean value of the new image histogram.
